# Non-Bacillary Croup

**Published:** 1900-03

**Authors:** J. O. Symes

**Affiliations:** Assistant Physician, Bristol General Hospital


### NON-BACILLARY CROUP.

BY

J. O. Symes, M.D., D.P.H. Lond.,

*Assistant Physician, Bristol General Hospital.*

---

An acute membranous affection of the fauces extending to the larynx and trachea, excited by agencies other than the Klebs-Löffler bacillus, has been variously described as "non-infective croupous angina," "pseudo-diphtheria," and "non-bacillary croup." The use of the word "diphtheria" in this condition, although etymologically correct, is to be deprecated. It should be applied only to those cases in which the diphtheria bacillus is present, other conditions being grouped under the head of non-bacillary croup.

Various agencies may excite a membranous condition of the fauces. Thus it may be caused by burns or scalds, by chemical irritants, by the use of the cautery, or by the invasion of the tissues by micro-organisms. In the cases detailed in the present paper, the first three of the above-mentioned causes could be eliminated, and as repeated examinations failed to reveal the presence of the Klebs-Löffler bacillus, they may be classed as true cases of non-bacillary croup of coccal origin. In undoubted cases of diphtheritic croup it frequently happens that bacteriological examination may give negative results as regards the Klebs-Löffler bacillus, owing to the swab being imperfectly inoculated, to the medium being unsuitable, or to the throat having been just previously irrigated with some strong antiseptic solution. For this reason I have only brought forward cases in which the examination of the membrane was made on several occasions, the cultures made on various media, and in some cases examined by more than one observer.

McBride and some other authors do not apparently recognise the condition of non-bacillary croup. McBride says: "It is extremely probable that most cases of fatal croup are in reality diphtheritic";[1] and again, "Most physicians will probably consider all cases of acute sore throat in which membranous exudation appears on the soft palate as diphtheritic."[2] Others, amongst whom I may mention Schech, Semon, Watson Williams, and Lennox Browne, recognise the condition; but I am not aware that a series of cases, in which careful bacteriological examination has been made, has yet been recorded, and for this reason I have considered the following cases[3] worthy of publication:—

1.—G. H., male, aged 1 year and 10 months, was admitted to the Children's Hospital, March 9th, 1898, with marked laryngeal obstruction, due to membranous exudation. Tracheotomy was performed and antitoxin injected. Repeated examinations of the exudate, by myself, revealed the presence of diplococci, streptococci, and staphylococcus aureus only. The same organisms were found by Dr. D. S. Davies (M.O.H.) in cultures made on March 11th and 18th and April 6th. The child was discharged cured on April 23rd.

[1] *Diseases of the Throat, Nose, and Ear*, 1892, p. 108. [2] *Ibid.*, p. 122.

[3] I have to thank Drs. Shaw, Prowse, Waldo, Elliott, and Rogers, for permission to publish notes of their cases.

2.—W. A. C., aged 3 years and 9 months, was admitted to the Children's Hospital on March 5th, 1899. There was nasal discharge, much membrane in the fauces, tonsils, uvula, and laryngeal obstruction necessitating immediate tracheotomy. The glands at the angle of the jaw were much swollen. Temperature 103° F. Antitoxin was injected. Direct examination of the exudate on stained films showed diplococci, and bacilli not of the Klebs-Löffler type. Cultures on Kanthack's medium showed streptococci and staphylococcus albus. Cultures by Dr. Davies on the same medium and on agar, streptococci only. The tracheotomy tube was removed on the fifth day, and the child discharged cured on March 31st.

3.—J. T., aged 10 years. Admitted to Children's Hospital March 10th, 1899. Nasal discharge, membrane on both tonsils, laryngeal obstruction, enlarged glands at angle of jaw. Several pieces of membrane were coughed up, and tracheotomy was necessary on March 11th. On the 12th, there was return of liquids through the nose. Some membrane was coughed up through the tube, which was kept in until March 15th. Recovery was uninterrupted. At no time was albumin present in the urine.

Direct microscopical examination of films prepared from the membrane on the first day showed strepto- and diplo-cocci, and long bacilli not of the Klebs-Löffler type, and cultures on agar showed the same organisms with the addition of staphylococci.

Dr. Davies, from cultures on Kanthack's medium, found staphylococci, and bacilli (non-diphtheritic).

4.—N. C., aged 2 years, was admitted to the Bristol Royal Infirmary on March 8th, 1898. On the previous day there had been convulsions and hoarseness, and on admission there was nasal discharge, membrane on tongue, tonsils, and pharynx, and signs of laryngeal obstruction. The glands at the angle of the jaw were enlarged. Broncho-pneumonia. Temperature 102.8° F. On March 9th tracheotomy was performed. Death occurred on March 10th. At the *post-mortem* examination there was found membrane throughout the whole respiratory track as far down as the bronchi, and patches of broncho-pneumonia. Cultures taken from the throat during life, both by Dr. Davies and myself, failed to show any diphtheria bacilli, the organisms present being staphylococci and various bacilli. Cultures made from a portion of membrane detached after death showed staphylococcus albus and citreus, whilst from the consolidated lung strepto- and staphylo-cocci were obtained.

5.—A. N., female, aged 4, was admitted to the Royal Infirmary on September 4th, 1898. This was the fifth day of the illness, and the laryngeal obstruction was such as to necessitate immediate tracheotomy. The pharynx, tonsils, and base of the tongue were covered with grey sloughy membrane, and several pieces of membrane were coughed up through the tracheotomy tube. Antitoxin was injected. Death ensued three hours after admission. At the *post-mortem* examination broncho-pneumonia was found, and extension of the membrane as far as the vocal cords. Cultures from and direct examination of the membrane after death showed only strepto- and staphylo-cocci.

6.—A. G., female, aged 4 years. Admitted to the Children's Hospital on August 10th, 1898. On this, the tenth day of the illness, there was laryngeal obstruction, and membranous exudation covered the fauces and soft palate. Temperature 99°. Antitoxin was administered, and on August 11th tracheotomy was performed. On August 13th a large slough came away from the throat; there was regurgitation of food through the nose and suppression of urine, and death ensued.

Cultures taken from the throat during life failed to reveal the presence of diphtheria bacilli, the only organisms present being diplo- and strepto-cocci.

7.—M. F. B., female, aged 4 years, was admitted to the Children's Hospital on April 21st, 1898, suffering from membranous pharyngitis and laryngitis with broncho-pneumonia. There was laryngeal stridor and much in-drawing of the lower intercostal spaces. Antitoxin was administered. Temperature 102°. Tracheotomy performed April 23rd. Child died April 25th. Repeated bacteriological examinations of the throat exudation showed staphylococcus albus and streptococci only.

8.—This was a case which cannot strictly be called non-bacillary. The laryngeal obstruction was such as to necessitate immediate intubation, followed by tracheotomy, and an enormous cast of trachea and bronchi to the fourth and fifth divisions was coughed up. The patient was a child aged 4, and died on the second day. Swabbings from the throat and rubbings from the membrane showed fusiform bacilli and spirals in great numbers. Cultures made both before and after death showed strepto- and staphylo-cocci only. This was undoubtedly a case of angina, due to the fusiform bacillus (Vincent).

Whilst there are many cases in which it is impossible, without a bacteriological examination, to distinguish between non-bacillary croup and true diphtheria, yet there are many clinical points on which the two diseases differ widely one from another. The onset of non-bacillary croup is much more sudden than that of diphtheria, and the temperature rises more quickly and to a higher point.

The membrane has not the dead white appearance of diphtheritic membrane. It is yellowish in colour, softer, less firmly matted together, and more easily detached from the underlying tissues. Placed in water the membrane swells up, losing its characteristic shape, probably owing to the large amount of mucus it contains. The surface from which the membrane has been detached is seldom bleeding or ulcerated, and may preserve its epithelium quite intact. Examined microscopically the membrane is found to consist of pus cells, leucocytes, and fibrin.

It is extremely difficult to get specimens of urine from young children such as are chiefly attacked by this disease, but it is noteworthy that in Case 3 the urine was free from albumin throughout the illness.

The paresis which leads to regurgitation of liquids through the nose is chiefly seen in those cases in which the soft palate has been heavily coated. It is seldom severe or of long duration. The voice is not affected, nor have I seen any other symptoms of paralysis. In Case 6 a complete loss of knee-jerk was noted.

Although it is probably true, as stated in some text-books, that most cases of fatal laryngitis are in reality diphtheritic, yet the above-mentioned cases show that non-bacillary croup is a not uncommon disease and has a very high mortality, as evidenced by the fact that of seven cases four died. Death is generally consequent upon the general septic infection or upon broncho-pneumonia.

The involvement of the larynx and trachea in this disorder must be far more common in young children than in adults. During fifteen months' residence at the London Fever Hospital I examined a large number of cases of non-diphtheritic membranous tonsillitis and pharyngitis occurring in adults in the initial stage of scarlet fever, but in no one of these was the larynx involved to such an extent as to necessitate tracheotomy.

*Etiology.*—If film preparations be made direct from the throat-swabbing, it will be found that a very large variety of organisms are present. In cultures upon agar, blood-serum, or Kanthack's medium, the forms which most generally develop are streptococci, staphylococci, and a large stout bacillus with rounded ends; and if the disease is not actually caused by one or other of these, the association is a very close one. It is not possible to distinguish between the inflammations caused by different organisms.

*Treatment.*—The exudation can be easily detached by means of a swab, and the local condition much improved by syringing the fauces with a solution of formalin (1 in 250) and by irrigating the nose with warm boracic acid solution. The dyspnœa is relieved by intubation or tracheotomy, but the tubes require constant and careful cleaning on account of the excessive secretion of mucus. In marked septicæmic cases it is possible that relief would follow the administration of anti-streptococcus serum. The constitutional treatment is identical with that employed in cases of diphtheria.

